# Exposure to Air Pollution and the Prevalence of Respiratory Symptoms in Northeast Ohio Farmworkers

**DOI:** 10.1007/s10903-025-01700-2

**Published:** 2025-05-20

**Authors:** Maeve G. MacMurdo, Veronica Dahlberg, Karen B. Mulloy, Jacqueline Curtis, Daniel A. Culver

**Affiliations:** 1 Cleveland Clinic, Cleveland, USA; 2 HOLA Ohio, Painesville, USA; 3 Case Western Reserve University, Cleveland, USA

**Keywords:** Agriculture, Farmworker health, Outdoor air pollution, Respiratory health

## Abstract

The burden of chronic respiratory disease among the Latino farmworker community remains poorly understood. We aim to evaluate the prevalence of self-reported respiratory symptoms in a cohort of Latino farmworkers and identify the association between occupational and environmental exposures and symptom burden. Utilizing participatory mapping in combination with satellite derived models of ambient air pollution exposure, we quantified estimated exposure to PM_2.5_ over the harvest season. Self-reported exposure to pesticides, crowded housing and tobacco were evaluated via survey, along with self-reported prevalence of respiratory symptoms. One third of our participants (33.7%) reported persistent respiratory symptoms. We did not identify an association between PM_2.5_ concentrations and self-reported respiratory symptoms. Loading and applying pesticides, and an increased ratio of workers per bedroom were associated with an increased risk of self-reported respiratory symptoms. Within our sample, respiratory symptoms were prevalent at a higher frequency than reported in national farmworker surveys. No association between estimated exposure to ambient air pollution and self-reported respiratory symptoms was identified.

## Background

The United States employs approximately 3 million migrant farmworkers [1]. Regardless of job duties, migrant farmworkers are at high risk for occupational injury, with a workplace fatality rate more than seven times that of the general United States workforce [2]. While the risk of workplace injury and fatality in the farmworker community is well recognized, the burden of chronic disease among migrant farmworkers in the United States remains poorly understood.

Migrant farmworkers are uniquely at risk for poor working conditions when compared to the general farmworker population. Working conditions are highly variable, and range from planting and harvesting, to orchard, nursery and cannery work. The vast majority of the three million farmworkers in the US are immigrants from Latin America [3]. While estimates vary, more than 1/3 of farmworkers may be undocumented, and 65% have little or no English-speaking ability [3]. 

Outside of the migrant farmworker population, the respiratory health hazards associated with occupational exposures in farming are well recognized. Occupational exposure to animal protein and grain dust have been linked to an increased risk of asthma, chronic bronchitis and fibrotic lung disease [4–6]. While data remain mixed, exposure to pesticides has been associated with an increased risk of self-reported wheeze, physician diagnosed asthma and impairments in lung function [7–11]. 

Despite this, little is known about the respiratory health of the migrant farmworker community. Among a cohort of New York farmworkers completing OSHA respirator FIT testing, self-reported respiratory symptoms were reported by less than 3% of participants, though this data has significant limitations- workers who do endorse symptoms face a significant risk of unemployment [12]. By comparison, surveys in a cohort of North Carolina based farmworkers living in employer provided housing found a high prevalence of cough, wheeze, and rhinitis [13]. Because farmworkers face multiple barriers to accessing healthcare, research into the prevalence of documented respiratory illness (such as asthma or COPD) has major limitations. In this context, the prevalence of self-reported systems represents an important surrogate for the true frequency of respiratory disease [14, 15]. 

Exposure to ambient air pollution, particularly fine particulate matter (PM_2.5_) may be an important driver of respiratory disease in farmworkers [16, 17]. The negative impacts of particulate air pollution on health are clear [18–20]. To date, there is limited literature investigating the health impacts of air pollution on farmworker health. In the setting of climate change, the exposure to PM_2.5_ faced by farmworkers is anticipated to worsen [21]. Quantifying exposure to PM_2.5_ faced by farmworkers has significant challenges- particularly in rural regions, stationary monitoring of PM_2.5_ is either limited or entirely absent.

To overcome these challenges, we utilize participatory mapping combined with satellite derived estimates of ambient PM_2.5_ to investigate the impact of exposure to ambient air pollution, in combination with other common farmworker associated exposures on self-reported respiratory symptoms in a cross-sectional sample of Latino farmworkers. Our primary hypothesis was that farmworkers exposed to higher levels of ambient PM_2.5_ would experience increased respiratory symptom burden, and that respiratory symptoms would be common among our participants.

## Methods

In collaboration with our community partner farmworkers currently employed in agricultural work in the greater Northeast Ohio area were recruited for participation throughout 2022 and early 2023. A convenience sampling framework was used for recruitment, leveraging a combination of community events and field data collection. A recruitment target of 100 workers was set to ensure sampling of a diverse range of workers within the local community. Farmworkers were eligible for participation if they were greater than 18 years of age and actively employed in agriculture. Recruitment occurred throughout July-October and January-March harvest periods and was completed with guidance from our community partner about the harvest season and timing of workers contracts in the area. While there is significant variation in job tasks by employer, there are not significant differences in task or employment opportunity by gender, and job tasks are similar across both sampling periods.

Participants were asked to complete a short survey in either Spanish or English. Self-reported respiratory symptoms were assessed utilizing a modified version of the National Health and Nutrition Examination 2011–2012 and national agricultural workers survey 2002–2003 respiratory health questionnaire to allow for comparison against national trends [22, 23]. Participants were subsequently queried about housing conditions over the prior 12 months, work in a field in which pesticide had been sprayed within the past 12 months, application or active use of pesticides within the past 12 months, and prior episodes of pesticide poisoning. Participants were queried regarding their past pulmonary history, including prior diagnoses of asthma, chronic obstructive pulmonary disease, prescriptions for respiratory therapy and access to medical care for respiratory symptoms.

Following survey completion, participants utilized a paper map to mark the approximate location of the farm or site where they were currently employed, along with any other sites or locations where they had worked an agricultural job during the 12-month period prior to participation. To minimize the risk of potential loss of privacy or employer retaliation, participants were not asked to give the name of their employer. Visa status was also not queried. This study was approved as exempt by the Cleveland Clinic Institutional Review Board (IRB number 22–430).

Following completion of the paper map, location data were subsequently digitized and mapped at the point level using ArcGIS Pro (V3.1.0). Concentration of PM_2.5_ were estimated at the 1 km radius around participant reported work locations for the 12 months prior to recruitment, based on the harvest period (July-October or January-March) during which recruitment occurred utilizing satellite derived models of PM_2.5_ developed by Van Donklear et al. [24] These models utilize satellite derived estimates of aerosol optical depth (AOD) in combination with GEOS-chem chemical transport models, calibrated against ground-based observations of PM_2.5_ to generate estimates of PM_2.5_ at a fine spatial scale across the continuous United States, overcoming the limitations of rural stationary monitor estimates of exposure.

Utilizing R version 4.3.1 descriptive statistics for the cohort were generated. Logistic regression was utilized to investigate the association between mean exposure to PM_2.5_ and self-reported respiratory symptoms (defined as self-reported cough, wheeze, chest tightness, shortness of breath or persistent rhinitis over the past 12-month period), and the association between self-reported respiratory symptoms and self-reported exposure to other potential respiratory irritants, specifically, tobacco use, crowded housing, pesticide application, and working in a field where pesticides had been recently sprayed or applied. All models were adjusted for age, and years of agricultural employment. Stratified analysis by gender was completed.

## Results

A total of 96 farmworkers completed both location mapping and the study questionnaire. The vast majority were male, with only 13.5% of the participants identifying as female. (Table 1) All identified as Latino. The median age was 28 (IQR 24–38), with a median 4 years of prior agricultural work (IQR 2–6). Tobacco use was uncommon. Only 16.9% of participants reporting having ever smoked tobacco, with 8.4% of participants reporting active tobacco use. Among those who did smoke, mean packs smoked per day was comparatively low (0.36 packs/day), with several reporting tobacco use once a month or less.

**Table 1:** Prevalence of self-reported symptoms and tobacco use by participant gender, with comparison to National agricultural worker survey respiratory symptom reporting

Variable	Male (*n* = 83)	Female (*n* = 13)	National Agricultural Worker survey (2003) [22]
Cough (n)	10.8% (9)	28.5% (5)	1%
Wheezing at rest or with activity (n)	13.3% (11)	46.7% (6)	3%
Daily phlegm (n)	10.8% (9)	15.4% (2)	1%
Chest tightness (n)	9.6% (8)	30.8% (4)	4%
Rhinitis (n)	16.9% (14)	53.8% (7)	7%
Ever smoker*	16.9% (14)	0% (0)	33%

* Defined as 100 cigarettes or more over a lifetime

Approximately one third (33.7%) of participants reported experiencing respiratory symptoms within the past year, with rhinitis being the most reported symptom (21.8% of participants), followed by cough (14.5%) (Table 1). Despite the fact that 33% of participants reported experiencing either chest tightness, wheezing at rest or with exertion, only one participant had a confirmed diagnosis of asthma, a prevalence significantly lower than the national average [25]. Only 3% of participants had seen a doctor within the past year. 11.4% of participants reported being worried about the impact of air quality on their health. Female participants were more likely to report experiencing respiratory symptoms (76.9% versus 28.9%, *P* = 0.002) and more likely to report being so sick that they were unable to work (30.7% versus 0.01%, *P* < 0.001).

An association between participant age and respiratory symptoms was not identified (unadjusted OR 0.98, CI 0.94–1.02, *p* = 0.39), or between duration of agricultural employment and the prevalence of respiratory symptoms (unadjusted OR 0.92, CI 0.84-1, *P* = 0.05).

Exposure to pesticides was common, with 15% of participants reporting spraying, loading or applying pesticide products within the past year, and 68% of participants reporting working in a field where pesticides had been recently sprayed or applied within the past year. 7% of participants reported having experienced an episode of pesticide poisoning at any point in their working career. Though not statistically significant, there was a trend towards a relationship between self-reported respiratory symptoms and working in a field where pesticides had been sprayed within the past 12 months (unadjusted OR 2.19, CI 0.88–5.89, *P* = 0.10). No change in this relationship was seen when stratified by gender. In addition, workers who reported working in a field where pesticides had been recently sprayed reported a higher frequency of persistent cough (21% versus 3%, *P* = 0.04) and chest tightness (19% versus 0%, *P* = 0.02). Workers who reported loading or applying pesticides within the past 12 months were more likely to report respiratory symptoms (unadjusted OR 4.10, CI 1.28–14.5, *P* = 0.02), This relationship was not significant when adjusted for age and years of agricultural employment, though significant differences were seen with evaluation stratified by gender. (Table 2).

**Table 2:** Risk of self-reported respiratory symptoms by self-reported exposure

Variable	Unadjusted OR (CI)	*P*	Adjusted OR* (CI)	*P*	Unadjusted OR stratified by male gender (CI)	*P*
Mean PM2.5	0.76 (0.23–2.41)	0.63	0.62 (0.17–2.13)	0.45	0.61 (0.14–2.53)	0.50
Pesticide spraying	2.19 (0.0.88–5.89)	0.10	2.16 (0.85–5.95)	0.11	2.79 (0.98–9.34)	0.07
Pesticide loading	4.10 (1.28–14.5)	0.02	3.22 (0.88–12.67)	0.07	4.58 (1.18–19.7)	0.03
Current tobacco use	1.38 (0.26–6.64)	0.68	1.16 (0.2–5.99)	0.85	1.93 (0.36–9.48)	0.42
Worker-to-Bedroom Ratio	2.12 (1.38–3.50)	0.001	2.25 (1.38–3.96)	0.002	2.03 (1.23–3.48)	0.004

* Adjusted for participant age and years of agricultural employment

More than half of participants (65%) reported living in crowded housing, with two or more workers per bedroom. Workers who reported living in crowded housing were younger with a mean age of 27.6 years (SD 7.8) compared with those living in non-crowded housing (37.8 years, SD 10.6, *P* < 0.001), and had less experience in agricultural work (4.16 years versus 7.81 years, *P* < 0.001). An increase in the number of workers sharing a bedroom was associated with an increased risk of respiratory symptoms (unadjusted OR 2.19, CI 1.38–3.50, *P* < 0.001), which remained significant when adjusted for age and years in agricultural employment.

Mean exposure to PM_2.5_ across all participants was estimated at 7.23 mg/m^3^ (SD 6.99–7.4 mg/m^3^). No significant difference in estimated ambient PM_2.5_ concentrations was seen between workers surveyed during the Summer (mean 7.307 mg/m^3^) and Fall (mean 7.164 mg/m^3^) harvest period. A relationship between self-reported respiratory symptoms and estimated PM_2.5_ exposure was not identified (unadjusted OR 0.76 CI 0.23–2.41, P 0.63).

## Discussion

Within a sample of actively employed farmworkers working throughout Northeast Ohio, respiratory symptoms were relatively common, with 33% of participants reporting experiencing one or more symptoms concerning for underlying reactive airway disease, specifically chest tightness, wheezing or wheezing with activity over the preceding 12 months. This is significantly higher than has been reported in previous national studies of migrant farmworker health (Table 2) [22, 26]. This may reflect the differences in exposure, or differences in participant recruitment- notably, the National Agricultural Worker Survey does not include H2-A guest workers, who make up an increasing proportion of the migrant farmworker labor force in the United States [27]. Because workers employed through the H2-A program are reliant on their employer for housing and transport, they may be more vulnerable to poor housing and working conditions.

Kearney et al. have previously identified a similar prevalence of respiratory symptoms in a cohort of North Carolina migrant farmworkers, residing in agricultural labor camps [13]. Similar to our participant population, a significant proportion of farmworkers surveyed reported crowded housing, and poor housing conditions [13]. Residential exposure to mold, pests and poor indoor air quality has been associated with an increased risk of asthma across multiple populations outside of the migrant farmworker community [28–31].

A significant difference in the rate of all symptom reporting was seen by participant gender, with female participants reporting higher frequencies of cough, wheeze, chest tightness and rhinitis, despite similar patterns of occupational exposure. This difference may reflect barriers to reporting respiratory symptoms in our male participants rather than biological differences in the health impacts of our occupational exposures. Workers who develop an illness that makes them unable to work are likely to face both significant financial harm, and potential loss of visa status. Despite our efforts to maximize participant privacy and minimize collection of identifying information, this lack of worker protection likely impacts symptom reporting.

While participants reported experiencing a range of exposures potentially linked to respiratory health outcomes, only the loading and application of pesticides, and an increase in the number of workers per bedroom was associated with an increased risk of self-reported respiratory symptoms. Exposure to ambient air pollution was not associated with an increased risk of respiratory symptoms. The lack of meaningful relationship may reflect the fact that overall estimated exposure to PM_2.5_ was comparatively low, with mean exposure for both cohorts of participants falling below the limits set by National Ambient Air Quality Standards [32]. Alternatively, this may reflect barriers in our ability to fully estimate the exposure to ambient air pollution experienced by farmworkers utilizing a satellite-based approach.

Satellite derived estimates of air pollution exposure utilize a surrogate marker of PM_2.5_, the Aerosol Optical Depth (AOD). While the model utilized in our work combines measurements of AOD with ground based PM_2.5_ monitoring to enhance accuracy of measurements, satellite derived measures of AOD may not truly reflect the exposure to PM_2.5_ workers experience at “breathing height”, particularly in the setting of agricultural labor, where individual tasks such as loading, sweeping, and harvesting may result in significant, localized release of particulate matter. Additionally, particularly over the winter months, cloud cover within Northeast Ohio poses a significant barrier to satellite derived estimates. There are significant barriers to utilizing personal air quality monitoring to better understand the air pollution exposure faced by the farmworker community, including concern for worker privacy, the need for device calibration, and the physical nature of this work. However, these barriers are not insurmountable- Moran et al. were able to utilize personal air monitoring devices to quantify the PM_2.5_ exposure faced by produce workers- identifying substantial short-term release of particulate matter during work tasks [16]. Additional research that leverages both large scale estimates of exposure in combination with personal exposure measurement and objective measurement of impairment is needed.

## New Contributions to the Literature

Working in partnership with a local community advocacy group we identified a prevalence of respiratory symptoms within a sample of North East Ohio farmworkers, combined with widespread exposure to potential drivers of occupational lung disease. This highlights the need for further work to better understand the respiratory health of the farmworker community across the United States, focusing on both the health impacts of occupational exposures faced by farmworkers, and the impact of financial insecurity, discrimination, and poor housing quality on worker health.

## Data Availability

The data that support the findings of this study are not openly available due to participant privacy and are available from the corresponding author upon reasonable request. Estimates of ambient air pollution utilized in this study are publicly available and developed by Dr. Van Donkelaar et al. (Reference: Aaron van Donkelaar, Melanie S. Hammer, Liam Bindle, Michael Brauer, Jeffery R. Brook, Michael J. Garay, N. Christina Hsu, Olga V. Kalashnikova, Ralph A. Kahn, Colin Lee, Robert C. Levy, Alexei Lyapustin, Andrew M. Sayer and Randall V. Martin (2021). Monthly Global Estimates of Fine Particulate Matter and Their Uncertainty Environmental Science & Technology, 2021, 10.1021/acs.est.1c05309)
